# Peripheral Blood Invariant Natural Killer T Cells of Pig-Tailed Macaques

**DOI:** 10.1371/journal.pone.0048166

**Published:** 2012-10-23

**Authors:** Xiangming Li, Patricia Polacino, Raquel Garcia-Navarro, Shiu-Lok Hu, Moriya Tsuji

**Affiliations:** 1 HIV and Malaria Vaccine Program, Aaron Diamond AIDS Research Center, Affiliate of the Rockefeller University, New York, New York, United States of America; 2 Washington National Primate Research Center, University of Washington, Seattle, Washington, United States of America; 3 Department of Pharmaceutics, University of Washington, Seattle, Washington, United States of America; Karolinska Institutet, Sweden

## Abstract

In humans, invariant natural killer T (*i*NKT) cells represent a small but significant population of peripheral blood mononuclear cells (PBMCs) with a high degree of variability. In this study, pursuant to our goal of identifying an appropriate non-human primate model suitable for pre-clinical glycolipid testing, we evaluated the percentage and function of *i*NKT cells in the peripheral blood of pig-tailed macaques. First, using a human CD1d-tetramer loaded with α-GalCer (α-GalCer-CD1d-Tet), we found that α-GalCer-CD1d-Tet^+^ CD3^+^ *i*NKT cells make up 0.13% to 0.4% of pig-tailed macaque PBMCs, which are comparable to the percentage of *i*NKT cells found in human PBMCs. Second, we observed that a large proportion of Vα24^+^CD3^+^ cells are α-GalCer-CD1d-Tet^+^CD3^+^ *i*NKT cells, which primarily consist of either the CD4^+^ or CD8^+^ subpopulation. Third, we found that pig-tailed macaque *i*NKT cells produce IFN-γ in response to α-GalCer, as shown by ELISpot assay and intracellular cytokine staining (ICCS), as well as TNF-α, as shown by ICCS, indicating that these *i*NKT cells are fully functional. Interestingly, the majority of pig-tailed macaque *i*NKT cells that secrete IFN-γ are CD8^+^ *i*NKT cells. Based on these findings, we conclude that the pig-tailed macaques exhibit potential as a non-human animal model for the pre-clinical testing of *i*NKT-stimulating glycolipids.

## Introduction

Natural killer T (NKT) cells are a unique subset of lymphoid cells that express both a T cell antigen receptor (TCR) and NK1.1 (NKR-P1 or CD161c), a C-lectin-type NK receptor [1], [2]. A significant proportion of NKT cells express semi-invariant TCRs encoded by Vα24 and Jα18 gene segments in humans and Vα14 and Jα18 gene segments in mice, and these cells have been designated invariant NKT (*i*NKT) cells [3]. In humans, *i*NKT cells represent a small but significant proportion (0.01%–0.5%) of PBMCs with a high degree of variability [4], [5]. Upon activation, *i*NKT cells rapidly secrete both Th1 and Th2 cytokines *in vivo* and induce a series of cellular activation events leading to the activation of innate immune cells, such as NK cells and dendritic cells (DCs), as well as the stimulation of adaptive immune cells, such as B and T cells [6]–[12]. In addition, upon stimulation, *i*NKT cells, like NK cells, display cytotoxic activity mediated by Fas, perforin, granzyme A/B, and granulysin [13], [14]. *i*NKT cells have also been shown to display anti-tumor activity [15], [16], mediate therapeutic effects against autoimmune diseases [17]–[20], and promote protection against certain infectious agents [21]–[24].

CD1d molecules and *i*NKT cells are conserved between mice and humans [25]. Accordingly, mouse models have been extensively used to study the biological activity of CD1d-binding, *i*NKT cell-stimulating glycolipids, and the phenotypes and functions of *i*NKT cells [1], [26]. However, these studies have indicated substantial differences in the specificity, frequency, and function of CD1d and *i*NKT cells between the two species. Because of this, some studies have investigated the frequency, phenotype, and function of *i*NKT cells derived from non-human primates, including pig-tailed macaques, and found similar percentages and high variability of *i*NKT cells between monkeys and humans [27]–[30]. These studies have also indicated that the phenotypes and functions of monkey *i*NKT cells are significantly different among different macaque species [27]–[30]. Pig-tailed macaques have been used as animal models to study a number of human diseases, such as *Chlamydia trachomatis* [31]–[33] and HIV-1 infection [34], [35]. In this study we sought to characterize in greater detail the base line frequency, specificity, and function of *i*NKT cells in pig-tailed macaques and address whether pig-tailed macaques could be used as an animal model for the pre-clinical testing of various *i*NKT cell-stimulating ligands.

**Figure 1:**
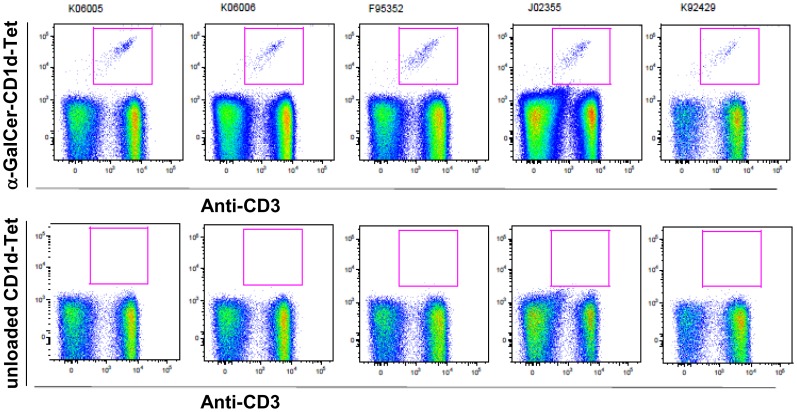
α-GalCer-CD1d-Tet+CD3+ cells among PBMCs from pig-tailed macaques. Peripheral blood mononuclear cells (PBMCs) obtained from each pig-tailed macaque were incubated with anti-CD3-PerCP together with α-GalCer-loaded human CD1d-tetramer-PE (α-GalCer-CD1d-Tet-PE) in upper panel or unloaded human CD1d-tetramer-PE as a negative control in lower panelData were analyzed using Flowjo software (Tree Star). For all figures, the data represent one of three similar experiments.

## Materials and Methods

### Animals

Pig-tailed macaques (*M. nemestrina*) were used in this study. All animals were negative for simian immunodeficiency virus (SIV) and simian T-cell lymphotropic virus type 1 (STLV-1) by serology as well as simian type D retrovirus by serology and polymerase chain reaction (PCR). Peripheral blood was collected by venipuncture under anesthesia. All animals used in this study were housed and cared for according to the Guide for the Care and Use of Laboratory Animals at the Washington National Primate Research Center (WaNPRC), an institution accredited by the Association for Assessment and Accreditation of Laboratory Animal Care International. The animal quarters are maintained at 75–78°F with controlled air humidity and quality. Commercial monkey chow was fed to the animals once daily, and drinking water was available at all times. Daily examinations and any medical care were provided by the WaNPRC veterinary staff in consultation with the clinical veterinarian. All experimental procedures were approved by the Institutional Animal Care and Use Committee at the University of Washington and conducted in compliance with the Public Health Services Policy on Humane Care and Use of Laboratory Animals (http://grants.nih.gov/grants/olaw/references/PHSPolicyLabAnimals.pdf). The animals were kept under deep sedation with ketamine HCl at a dose of 10–15 mg/kg intramuscularly to alleviate any pain and discomfort during blood draws. An animal technician or veterinary technologist monitored the animals while under sedation.

**Figure 2:**
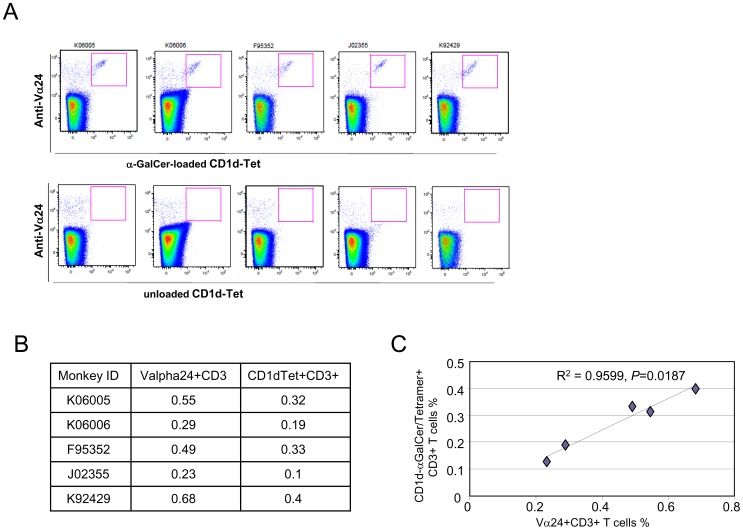
Correlation between Vα24+CD3+ cells and α-GalCer-CD1d-Tet+CD3+ cells among PBMCs from pig-tailed macaques. (A) One million PBMCs were incubated with anti-Vα24-FITC together with α-GalCer-CD1d-Tet-PE or unloaded CD1d-Tet-PE as a negative control then subjected to flow cytometric analysis, as described in Fig. 1. (B) The percentages of Vα24+CD3+ cells and α-GalCer-CD1d-Tet^+^CD3^+^ cells among PBMCs from each pig-tailed macaque are listed. (C) The percentage of Vα24^+^CD3^+^ cells and the percentage of α-GalCer-CD1d-Tet^+^CD3^+^ cells among PBMCs from each pig-tailed macaque are scatter-plotted to evaluate the correlation between these variables, and a linear regression analysis was applied.

### Preparation of Peripheral Blood Mononuclear Cells (PBMCs)

PBMCs were isolated from buffy coats by Ficoll-Hypaque density gradient separation. Erythrocytes were removed by osmotic lysis in ACK lysing buffer (Life Technologies, Grand Island, NY), and the remaining nucleated cells were washed twice with RPMI supplemented with 10% fetal calf serum (FCS).

**Figure 3:**
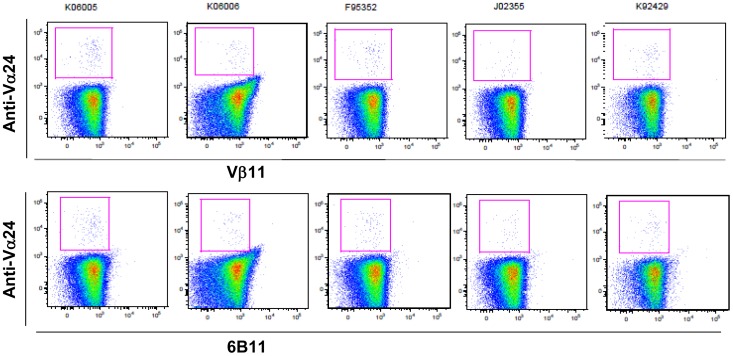
Vβ11 and 6B11 phenotypes of Vα24+ cells among PBMCs from pig-tailed macaques. One million PBMCs were incubated with anti-Vα24-PE together with either anti-Vβ11-FITC or 6B11-FITC followed by flow cytometric analysis, as described in Fig. 1.

### Antibodies, Glycolipid, and CD1d-tetramer

Anti-human antibodies known to cross-react with macaques were selected for this study. For flow cytometric analysis, we used anti-Vα24-PE (C15; Immunotech, Quebec, Canada), anti-Vα24-FITC (C15; Immunotech), anti-Vβ11-FITC (C21; Beckman Coulter, Brea, CA), anti-6B11-FITC (6B11; BioLegend, San Diego, CA), anti-CD3-perCp (SP34-2; BD Biosciences, San Jose, CA), anti-CD4-APC (SK3, BD Biosciences), anti-CD8-FITC (SK1, BD Biosciences), anti-CD8α-perCp (SK1, BD Biosciences), anti-CD8β-APC (2ST8.5H7, BD Biosciences), anti-IFN-γ-APC (4S.B3, Abcam, Cambridge, MA), and anti-TNF-α antibody-PE-Cy7 (MAb11, BioLegend). For ELISpot assay, we used anti-IFN-γ (clone: GZ-4, Mabtech, Mariemont, OH) and biotin-labeled anti-IFN-γ (clone: 7-B6-1, Mabtech). Lyophilized α-GalCer (Avanti Polar Lipid, Alabaster, AL) was reconstituted at 1 mg/ml with 100% DMSO then stored at −20°C. The α-GalCer-loaded human CD1d-tetramer conjugated to PE (α-GalCer-CD1d-Tet) was purchased from Proimmune Inc. (Sarasota, FL).

**Figure 4:**
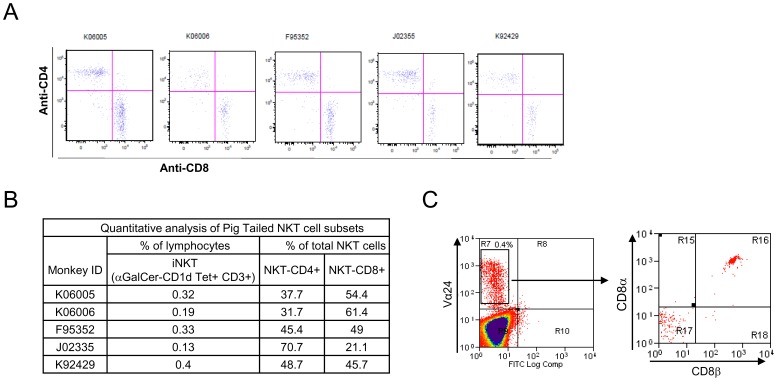
CD4/CD8 phenotype of α-GalCer-CD1d-Tet+CD3+ *i*NKT cells derived from pig-tailed macaques. (A) One million PBMCs were first incubated with α-GalCer-CD1d-Tet-PE and anti-CD3-PerCP. Cells were also stained with anti-CD4-APC and anti-CD8-FITC then subjected to flow cytometric analysis, as described in Fig. 1. Data represent one of two similar experiments. (B) The percentage of α-GalCer-CD1d-Tet^+^CD3^+^ cells among PBMCs and the percentage of CD4^+^ or CD8^+^ cells among α-GalCer-CD1d-Tet^+^CD3^+^ *i*NKT cells of each pig-tailed macaque are listed. (C) One million PBMCs were first incubated with anti-Vα24-PE then stained with CD8α-perCp anti-CD8β-APC and subjected to flow cytometric analysis, as described in Fig. 1.

### Flow Cytometric Analysis

For cell surface staining, 1×10^6^ PBMCs were incubated for 20 min at 4°C in FACs staining buffer in the presence of the antibody of interest. After washing twice, labeled cells were subjected to multicolor FACScan flow cytometry on a BD LSRII (Becton Dickinson, Franklin Lakes, NJ) using forward and side-scatter characteristics to exclude dead cells. Anti-mouse-Ig or anti-rat compensation particle sets were used for compensation purposes (BD Biosciences). The data were analyzed using Flowjo software (Tree Star, Ashland, OR).

**Figure 5:**
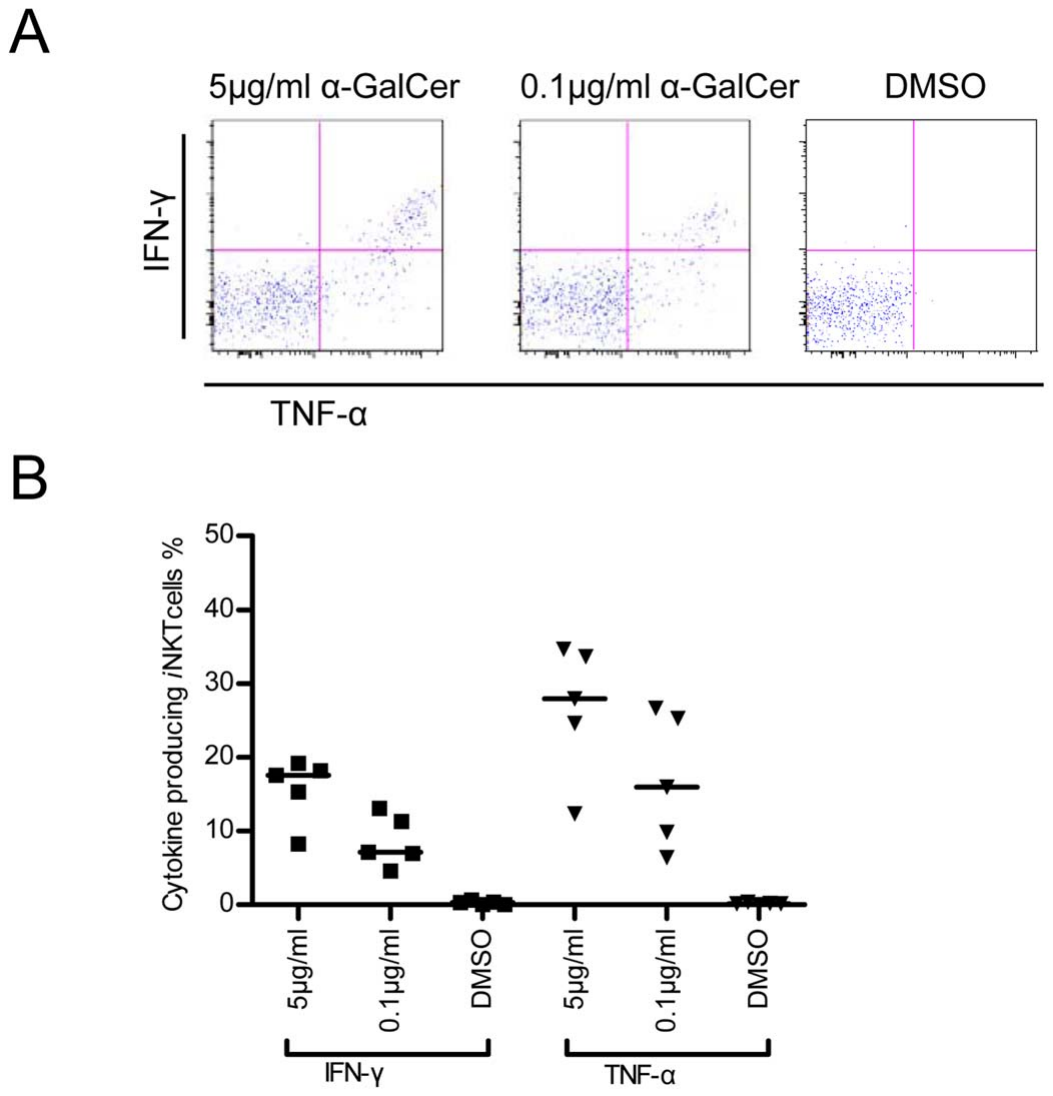
Secretion of IFN-γ and TNF-α by pig-tailed macaque *i*NKT cells upon stimulation with α-GalCer. One million PBMC cells were stimulated with 0.1 µg/ml or 5 µg/ml of α-GalCer followed by the addition of 5 µg/ml Brefeldin A for the last 4 hours of incubation. Invariant natural killer T (*i*NKT) cells were then gated with α-GalCer-CD1d-Tet^+^ and CD3^+^ followed by flow cytometric analysis. (A) Flow cytometric figure shows the pattern of IFN-γ and TNF-α expression by α-GalCer-activated, IFN-γ-secreting *i*NKT cells gated from PBMCs from one representative pig-tailed macaque. (B) The graph shows the percentages of *i*NKT cells secreting the respective cytokines among total *i*NKT cells derived from the PBMCs of five pig-tailed macaques.

**Figure 6:**
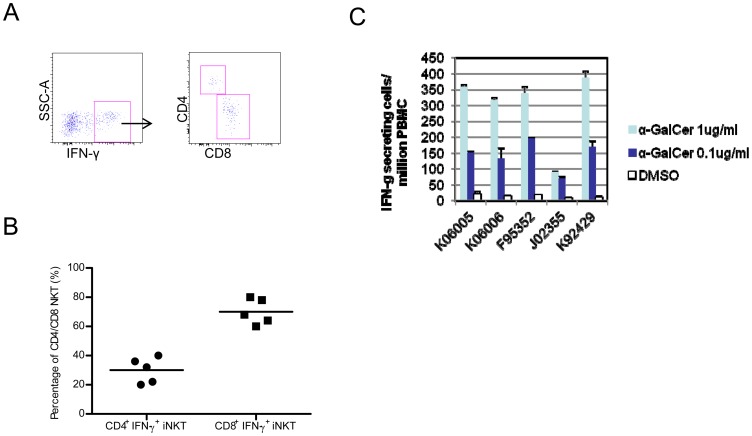
IFN-γ secreting pig-tailed macaque *i*NKT cells, as determined by intracellular cytokine staining and ELISpot assay. (A) Flow cytometric figure shows the pattern of CD4+ versus CD8+ expression by α-GalCer-activated, IFN-γ-secreting *i*NKT cells gated from the PBMCs from one representative pig-tailed macaque. v(B) The graph shows the percentages of CD4+ or CD8+ *i*NKT cells among total IFN-γ secreting *i*NKT cells derived from the PBMCs of five pig-tailed macaques. v(C) The relative number of α-GalCer-activated, IFN-γ-secreting *i*NKT cells among PBMCs. In this assay, 5×10^5^ pig-tailed macaque PBMC cells were stimulated with 0.1 µg/ml or 1 µg/ml of α-GalCer, and the relative numbers of IFN-γ-secreting cells were determined by an ELISpot assay. The results are expressed as the mean ± SD of triplicated wells.

### PBMCs Stimulation by α-GalCer

PBMCs were cultured in a 96-well U-bottom plate at 1×10^6^ cells/well in the presence of 5 µg/ml or 0.1 µg/ml of α-GalCer for 6 hours at 37°C followed by the addition of Brefeldin A (BioLegend) at 5 µg/ml for the last 4 hours of incubation. In a negative control group, cells were stimulated with medium containing 0.1% of DMSO vehicle.

**Figure 7:**
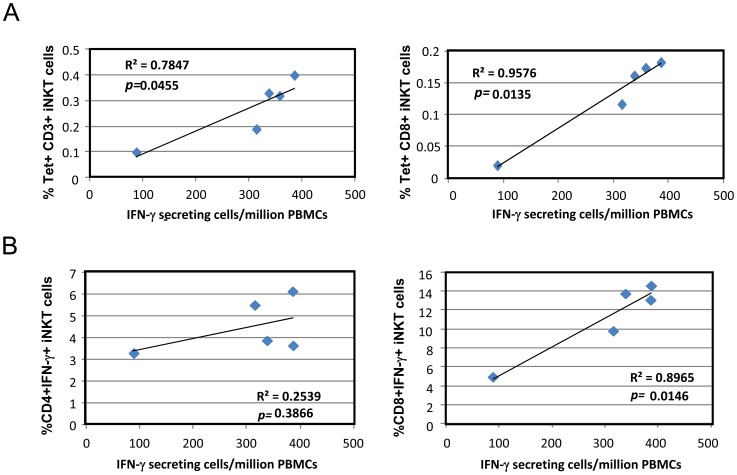
Correlation analysis of the surface phenotypes of pig-tailed macaque *i*NKT cells that secrete IFN-γ. (A) The relative numbers of IFN-γ secreting cells among PBMCs (ELISpot assay) and the percentage of α-GalCer-CD1d-Tet^+^CD3^+^ *i*NKT cells or CD8+ *i*NKT cells among PBMCs from each pig-tailed macaque (FACS analysis) are scatter-plotted to evaluate the correlation between these variables, and linear regression analysis was applied. (B) The relative numbers of IFN-γ secreting cells among PBMCs (ELISpot assay) and the percentage of CD4+ IFN-γ-secreting *i*NKT cells or CD8+ IFN-γ-secreting *i*NKT cells among PBMCs from each pig-tailed macaque (ICCS assay) are scatter-plotted to evaluate the correlation between these variables, and linear regression analysis was applied.

### Intracellular Cytokine Staining

For intracellular IFN-γ and TNF-α staining, the PBMCs were stimulated with α-GalCer, as described above. After stimulation, the cells were incubated with anti-CD3, anti-CD4, and anti-CD8 antibodies, as well as the human CD1d tetramer loaded with α-GalCer for 20 min. The cells were then fixed and permeabilized using the Cytofix/Cytoperm kit (BD Biosciences) following the manufacturer’s instructions. The permeabilized cells were stained with PE-Cy7-labeled anti-TNF-α and APC-labeled anti-IFN-γ antibodies for 30 min on ice in the dark. After washing twice, the cells were resuspended in staining buffer and analyzed by flow cytometric analysis. Acquisition and analysis were carried out by first gating for live cells by forward scatter (FSC) and side scatter (SSC) then subsequently gating for *i*NKT cells by positivity to CD3^+^ and α-GalCer-loaded CD1d tetramer^+^ among the live cells. IFN-γ^+^ and TNF-α^+^ cells were then further gated from the *i*NKT cells.

### IFN-γ ELISpot Assay

The total IFN-γ producing cells among the α-GalCer stimulated PBMC cells were determined by an ELISpot assay using the monkey IFN-γ ELISpot kit (Mabtech). Briefly, the Multiscreen HA ELISpot plate (Millipore, Billerica, MA) was first coated with anti-IFN-γ antibodies. Next, the PBMC cells from pig-tailed macaques were added at 5×10^5^ cells/well and stimulated with α-GalCer at 0.1 µg/ml or 1 µg/ml for 24 hours at 37°C. In the negative control groups, the cells were cultured with 0.1% DMSO. After washing five times, the plate was incubated with biotin-labeled anti-IFN-γ antibodies for 1 hour followed by incubation with avidin-HRP. Finally, the spots were developed with an AEC ELISpot substrate kit (BD Biosciences).

## Results and Discussion

Here, we aimed to characterize the properties of *i*NKT cells derived from pig-tailed macaques to determine whether the cells in this species exhibit similar properties to human *i*NKT cells. The goal of the study was to determine whether pig-tailed macaques represent an appropriate animal species for pre-clinical testing. We first determined the frequency of *i*NKT cells among PBMCs collected from pig-tailed macaques. To accomplish this, we identified *i*NKT cells by staining PBMCs with an α-GalCer-loaded human CD1d-tetramer (α-GalCer-CD1d-Tet). As shown in Fig. 1, we detected a distinct population of PBMCs that react with α-GalCer-CD1d-Tet, but not with the unloaded human CD1d-tetramer. The percentage of these α-GalCer-CD1d-Tet^+^ cells ranged from 0.13% to 0.4% of the total PBMCs (Fig. 2B). These results confirm those from a previously published study [30], and indicate that the percentage of *i*NKT cells in the peripheral blood of pig-tailed macaques is comparable to what has been observed in the peripheral blood of humans. To determine the correlation between α-GalCer-CD1d-Tet^+^ cells and Vα24^+^ cells, we co-stained pig-tailed macaque PBMCs with α-GalCer-CD1d-Tet and anti-Vα24 antibodies, as shown in Fig. 2A. We found that approximately two-thirds of the Vα24^+^ cells were α-GalCer-CD1d-Tet^+^ cells (Fig. 2B), and there was a strong positive linear correlation (*p* = 0.0187; R^2^ = 0.9599) between the percentages of the two subpopulations (Fig. 2C).

In humans, the majority of α-GalCer-CD1d-Tet^+^ *i*NKT cells have been shown to “co-express” an invariant Vα24-Jα18 chain and a semi-invariant Vβ11 chain [36]–[38]. Therefore, we sought to determine whether α-GalCer-CD1d-Tet^+^ *i*NKT cells derived from pig-tailed macaques also co-express Vα24 and Vβ11. Unfortunately, the anti-human Vβ11 antibody failed to cross-react with pig-tailed macaque *i*NKT cells, as has been shown with *i*NKT cells derived from other monkey species (Fig. 3) [27]–[29]. Furthermore, the 6B11 antibody, which is known to react with the CDR3 region of the Vα24-Jα18 chain, failed to react with Vα24^+^ cells derived from pig-tailed macaques (Fig. 3). Although the CDR3 region between the human and rhesus Vγ24 chain is almost identical (98% homology) [39], it is possible that the amino-acid sequence of the pig-tailed macaque Vα24 chain varies enough from the human Vα24 chain to lack the 6B11 epitope. This issue requires clarification and will be resolved in a future study.

We next determined the CD4 and CD8 phenotypes of pig-tailed macaque *i*NKT cells (Fig. 4A) and found that *i*NKT cells primarily consisted of the CD4+ and CD8+ subpopulations. The few remaining cells were double negative (DN)(Fig. 4B). All CD8^+^ *i*NKT cells were also found to be CD8αβ^+^ (Fig. 4C). These results confirmed an earlier study showing that pig-tailed macaque *i*NKT cells consist of a significant percentage of a CD4^+^ subpopulation [30]. Furthermore, the CD4/CD8 distribution is somewhat different from *i*NKT cells derived from other monkey species, which are largely made up of CD8^+^ cells [27]–[29]. More importantly, this distribution pattern resembles human *i*NKT cells, except DN *i*NKT cells are more abundant in humans [4], [5].

Regarding the functionality of *i*NKT cells, human *i*NKT cells activated by α-GalCer are known to secrete a myriad of cytokines, with CD8^+^ *i*NKT cells biased toward a Th1 phenotype, CD4^+^ *i*NKT cells predominantly secreting Th2 cytokines, and DN *i*NKT cells exhibiting an intermediate Th1/Th2 phenotype [40]. Non-human primate *i*NKT cells have been shown to display a similar function to human *i*NKT cells, but there are some differences among different species. For example, rhesus macaque *i*NKT cells secrete large amounts of TGF-β, IL-6, and IL-13, and modest levels of IFN-γ, whereas IL-10 secretion was negligible and no detectable IL-4 was observed [41]. However, sooty mangabey *i*NKT cells have been shown to secrete virtually all cytokines tested, including IFN-γ, TNF-α, IL-2, IL-13, and IL-10 [29]. In addition, their CD8^+^ NKT subpopulation produced a high amount of IFN-γ and expressed significantly higher levels of granzyme B and perforin [42].

To investigate the function of pig-tailed macaque *i*NKT cells in this study, we first measured the percentage of α-GalCer-activated *i*NKT cells secreting IFN-γ, TNF-α and IL-10, using an ICCS assay. As shown in Fig. 5, a significant percentage of pig-tailed macaque *i*NKT cells secreted TNF-α and/or IFN-γ, whereas they failed to secrete a significant amount of IL-10 (data not shown). We next analyzed the percentages of CD4^+^ and CD8^+^ *i*NKT cell subpopulations among the total IFN-γ-secreting *i*NKT cells. Although both CD4^+^ and CD8^+^ *i*NKT cells produced IFN-γ after stimulation with α-GalCer, the percentage of IFN-γ-secreting CD8^+^ *i*NKT cells was much higher than IFN-γ-secreting CD4^+^ *i*NKT cells (Fig. 6). Furthermore, the IFN-γ ELISpot assay showed that 100–400 per million PBMCs secreted IFN-γ in response to 1 µg/ml of α-GalCer (Fig. 6C). This result corroborates our ICCS assay and indicates that a significant number of *i*NKT cells among PBMCs secrete IFN-γ upon α-GalCer stimulation.

We then performed various correlation analyses and found a marginal correlation between the relative number of α-GalCer-activated cells secreting IFN-γ among PBMCs, as determined by ELISpot assay, and the percentage of total *i*NKT cells among PBMCs, as determined by FACS analysis (R^2^ = 0.7847, p = 0.0455) (Fig. 7A). However, the correlation became much stronger when we performed a correlation analysis between the relative number of α-GalCer-activated cells secreting IFN-γ among PBMCs and the percentage of CD8^+^ *i*NKT cells among PBMCs (R^2^ = 0.9576, p = 0.0135) (Fig. 7A). Interestingly, when we compared the relative number of IFN-γ-secreting cells among PBMCs and the percentages of IFN-γ-secreting CD8^+^ and CD4^+^ *i*NKT cells by ICCS assay, we found a strong correlation for the relative number of IFN-γ-secreting cells among PBMCs with IFN-γ-secreting CD8^+^ *i*NKT cells (R^2^ = 0.8965, p = 0.0146), but not with IFN-γ^ +^ CD4^+^ *i*NKT cells (R^2^ = 0.2559, p = 0.3866) (Fig. 7B). Thus, our current functional study demonstrates that the majority of pig-tailed macaque *i*NKT cells that secrete IFN-γ consist of CD8^+^ *i*NKT cells, although CD4^+^ *i*NKT cells can also produce Th1 cytokines, including IFN-γ and TNF-α.

We would like to emphasize that due to the lack of available antibodies that cross-react with pig-tailed macaque cells, we could only perform a limited study of the phenotype and function of pig-tailed macaque *i*NKT cells. Despite this difficulty, however, our current study demonstrates that the percentage of *i*NKT cells present in the peripheral blood of pig-tailed macaques is comparable to the *i*NKT cells found in human peripheral blood. Furthermore, similar to humans, a large proportion of Vα24^+^CD3^+^ cells are α-GalCer-CD1d-Tet^+^CD3^+^ *i*NKT cells, and almost half of these express CD4 molecules.

Together, these results highlight the properties of pig-tailed macaque *i*NKT cells, which resemble human cells to some degree. In light of previous successful research studies using pig-tailed macaques for certain human diseases [31]–[35], our study provides further evidence supporting the use of pig-tailed macaques in the pre-clinical testing of various *i*NKT cell-stimulating ligands. In particular, they may be useful for evaluating therapeutic and prophylactic measures across a myriad of human diseases in the future.

## References

[1] Bendelac A, Rivera MN, Park SH, Roark JH (1997) Mouse CD1-specific NK1 T cells: development, specificity, and function. Annu Rev Immunol 15: 535–562. 9143699 10.1146/annurev.immunol.15.1.535

[2] Godfrey DI, Hammond KJ, Poulton LD, Smyth MJ, Baxter AG (2000) NKT cells: facts, functions and fallacies. Immunol Today 21: 573–583. 11094262 10.1016/s0167-5699(00)01735-7

[3] Benlagha K, Weiss A, Beavis A, Teyton L, Bendelac A (2000) In vivo identification of glycolipid antigen-specific T cells using fluorescent CD1d tetramers. J Exp Med 191: 1895–1903. 10839805 10.1084/jem.191.11.1895 PMC2213523

[4] Vasan S, Poles MA, Horowitz A, Siladji EE, Markowitz M, et al (2007) Function of NKT cells, potential anti-HIV effector cells, are improved by beginning HAART during acute HIV-1 infection. Int Immunol 19: 943–951. 17702988 10.1093/intimm/dxm055

[5] Lucas M, Gadola S, Meier U, Young NT, Harcourt G, et al (2003) Frequency and phenotype of circulating Valpha24/Vbeta11 double-positive natural killer T cells during hepatitis C virus infection. J Virol 77: 2251–2257. 12525661 10.1128/JVI.77.3.2251-2257.2003 PMC140901

[6] Carnaud C, Lee D, Donnars O, Park SH, Beavis A, et al (1999) Cutting edge: Cross-talk between cells of the innate immune system: NKT cells rapidly activate NK cells. J Immunol 163: 4647–4650. 10528160

[7] Eberl G, MacDonald HR (2000) Selective induction of NK cell proliferation and cytotoxicity by activated NKT cells. Eur J Immunol 30: 985–992. 10760785 10.1002/(SICI)1521-4141(200004)30:4<985::AID-IMMU985>3.0.CO;2-E

[8] Kitamura H, Iwakabe K, Yahata T, Nishimura S, Ohta A, et al (1999) The natural killer T (NKT) cell ligand alpha-galactosylceramide demonstrates its immunopotentiating effect by inducing interleukin (IL)-12 production by dendritic cells and IL-12 receptor expression on NKT cells. J Exp Med 189: 1121–1128. 10190903 10.1084/jem.189.7.1121 PMC2193012

[9] Fujii S, Shimizu K, Smith C, Bonifaz L, Steinman RM (2003) Activation of natural killer T cells by alpha-galactosylceramide rapidly induces the full maturation of dendritic cells *in vivo* and thereby acts as an adjuvant for combined CD4 and CD8 T cell immunity to a coadministered protein. J Exp Med 198: 267–279. 12874260 10.1084/jem.20030324 PMC2194082

[10] Hermans IF, Silk JD, Gileadi U, Salio M, Mathew B, et al (2003) NKT cells enhance CD4+ and CD8+ T cell responses to soluble antigen *in vivo* through direct interaction with dendritic cells. J Immunol 171: 5140–5147. 14607913 10.4049/jimmunol.171.10.5140

[11] Kitamura H, Ohta A, Sekimoto M, Sato M, Iwakabe K, et al (2000) Alpha-galactosylceramide induces early B-cell activation through IL-4 production by NKT cells. Cell Immunol 199: 37–42. 10675273 10.1006/cimm.1999.1602

[12] Eberl G, Brawand P, MacDonald HR (2000) Selective bystander proliferation of memory CD4+ and CD8+ T cells upon NK T or T cell activation. J Immunol 165: 4305–4311. 11035065 10.4049/jimmunol.165.8.4305

[13] Taniguchi M, Nakayama T (2000) Recognition and function of Valpha14 NKT cells. Semin Immunol 12: 543–550. 11145860 10.1006/smim.2000.0270

[14] Gansert JL, Kiessler V, Engele M, Wittke F, Rollinghoff M, et al (2003) Human NKT cells express granulysin and exhibit antimycobacterial activity. J Immunol 170: 3154–3161. 12626573 10.4049/jimmunol.170.6.3154

[15] Kawano T, Cui J, Koezuka Y, Toura I, Kaneko Y, et al (1998) Natural killer-like nonspecific tumor cell lysis mediated by specific ligand-activated Valpha14 NKT cells. Proc Natl Acad Sci U S A 95: 5690–5693. 9576945 10.1073/pnas.95.10.5690 PMC20440

[16] Crowe NY, Smyth MJ, Godfrey DI (2002) A critical role for natural killer T cells in immunosurveillance of methylcholanthrene-induced sarcomas. J Exp Med 196: 119–127. 12093876 10.1084/jem.20020092 PMC2194015

[17] Hong S, Wilson MT, Serizawa I, Wu L, Singh N, et al (2001) The natural killer T-cell ligand alpha-galactosylceramide prevents autoimmune diabetes in non-obese diabetic mice. Nat Med 7: 1052–1056. 11533710 10.1038/nm0901-1052

[18] Sharif S, Arreaza GA, Zucker P, Mi QS, Sondhi J, et al (2001) Activation of natural killer T cells by alpha-galactosylceramide treatment prevents the onset and recurrence of autoimmune type 1 diabetes. Nat Med 7: 1057–1062. 11533711 10.1038/nm0901-1057

[19] Jahng AW, Maricic I, Pedersen B, Burdin N, Naidenko O, et al (2001) Activation of natural killer T cells potentiates or prevents experimental autoimmune encephalomyelitis. J Exp Med 194: 1789–1799. 11748280 10.1084/jem.194.12.1789 PMC2193586

[20] Singh AK, Wilson MT, Hong S, Olivares-Villagomez D, Du C, et al (2001) Natural killer T cell activation protects mice against experimental autoimmune encephalomyelitis. J Exp Med 194: 1801–1811. 11748281 10.1084/jem.194.12.1801 PMC2193577

[21] Kakimi K, Guidotti LG, Koezuka Y, Chisari FV (2000) Natural killer T cell activation inhibits hepatitis B virus replication *in vivo* . J Exp Med 192: 921–930. 11015434 10.1084/jem.192.7.921 PMC2193313

[22] Chackerian A, Alt J, Perera V, Behar SM (2002) Activation of NKT cells protects mice from tuberculosis. Infect Immun 70: 6302–6309. 12379709 10.1128/IAI.70.11.6302-6309.2002 PMC130331

[23] Kawakami K, Kinjo Y, Yara S, Koguchi Y, Uezu K, et al (2001) Activation of Valpha14(+) natural killer T cells by alpha-galactosylceramide results in development of Th1 response and local host resistance in mice infected with *Cryptococcus neoformans* . Infect Immun 69: 213–220. 11119508 10.1128/IAI.69.1.213-220.2001 PMC97874

[24] Gonzalez-Aseguinolaza G, Oliveira de C, Tomaska M, Hong S, Bruna-Romero O, et al (2000) α-GalCer-activated Vα14 NKT cells mediate protection against murine malaria. Proc Natl Acad Sci U S A 97: 8461–8466. 10900007 10.1073/pnas.97.15.8461 PMC26970

[25] Brossay L, Chioda M, Burdin N, Koezuka Y, Casorati G, et al (1998) CD1d-mediated recognition of an alpha-galactosylceramide by natural killer T cells is highly conserved through mammalian evolution. J Exp Med 188: 1521–1528. 9782129 10.1084/jem.188.8.1521 PMC2213408

[26] Taniguchi M, Harada M, Kojo S, Nakayama T, Wakao H (2003) The regulatory role of Valpha14 NKT cells in innate and acquired immune response. Annu Rev Immunol. 21: 483–513. 10.1146/annurev.immunol.21.120601.141057 12543936

[27] Motsinger A, Azimzadeh A, Stanic AK, Johnson RP, Van Kaer L, et al (2003) Identification and simian immunodeficiency virus infection of CD1d-restricted macaque natural killer T cells. J Virol 77: 8153–8158. 12829854 10.1128/JVI.77.14.8153-8158.2003 PMC161937

[28] Gansuvd B, Goodwin J, Asiedu CK, Jiang XL, Jargal U, et al (2008) Invariant natural killer T cells from rhesus macaque spleen and peripheral blood are phenotypically and functionally distinct populations. J Med Primatol 37: 1–11. 18199066 10.1111/j.1600-0684.2007.00222.x

[29] Rout N, Else JG, Yue S, Connole M, Exley MA, et al (2010) Paucity of CD4+ natural killer T (NKT) lymphocytes in sooty mangabeys is associated with lack of NKT cell depletion after SIV infection. PLoS One 5: e9787. 20352088 10.1371/journal.pone.0009787 PMC2844411

[30] Fernandez CS, Chan AC, Kyparissoudis K, De Rose R, Godfrey DI, et al (2009) Peripheral NKT cells in simian immunodeficiency virus-infected macaques. J Virol 83: 1617–1624. 19052081 10.1128/JVI.02138-08 PMC2643790

[31] Clements JE, Mankowski JL, Gama L, Zink MC (2008) The accelerated simian immunodeficiency virus macaque model of human immunodeficiency virus-associated neurological disease: from mechanism to treatment. J Neurovirol 14: 309–317. 18780232 10.1080/13550280802132832 PMC8797541

[32] Klatt NR, Canary LA, Vanderford TH, Vinton CL, Engram JC, et al (2012) Dynamics of simian immunodeficiency virus SIVmac239 infection in pigtail macaques. J Virol 86: 1203–1213. 22090099 10.1128/JVI.06033-11 PMC3255820

[33] Henning T, Fakile Y, Phillips C, Sweeney E, Mitchell J, et al (2011) Development of a pigtail macaque model of sexually transmitted infection/HIV coinfection using Chlamydia trachomatis, Trichomonas vaginalis, and SHIV(SF162P3). J Med Primatol 40: 214–223. 21781129 10.1111/j.1600-0684.2011.00488.x PMC3402033

[34] Thippeshappa R, Polacino P, Yu Kimata MT, Siwak EB, Anderson D, et al (2011) Vif substitution enables persistent infection of pig-tailed macaques by human immunodeficiency virus type 1. J Virol 85: 3767–79. 21289128 10.1128/JVI.02438-10 PMC3126129

[35] Hatziioannou T, Ambrose Z, Chung NP, Piatak M Jr, Yuan F, et al (2009) A macaque model of HIV-1 infection. Proc Natl Acad Sci U S A 106: 4425–4429. 19255423 10.1073/pnas.0812587106 PMC2657417

[36] Sanderson JP, Waldburger-Hauri K, Garzón D, Matulis G, Mansour S, et al (2012) Natural variations at position 93 of the invariant Vα24-Jα18 α chain of human *i*NKT-cell TCRs strongly impact on CD1d binding. Eur J Immunol 42: 248–255. 21956730 10.1002/eji.201141956

[37] Nicol AJ, Tazbirkova A, Nieda M (2011) Comparison of clinical and immunological effects of intravenous and intradermal administration of α-galactosylceramide (KRN7000)-pulsed dendritic cells. Clin Cancer Res 17: 5140–5151. 21653690 10.1158/1078-0432.CCR-10-3105

[38] Kunii N, Horiguchi S, Motohashi S, Yamamoto H, Ueno N, et al (2009) Combination therapy of in vitro-expanded natural killer T cells and alpha-galactosylceramide-pulsed antigen-presenting cells in patients with recurrent head and neck carcinoma. Cancer Sci 100: 1092–1098. 19302288 10.1111/j.1349-7006.2009.01135.x PMC11158111

[39] Kashiwase K, Kikuchi A, Ando Y, Nicol A, Porcelli SA, et al (2003) The CD1d natural killer T-cell antigen presentation pathway is highly conserved between humans and rhesus macaques. Immunogenetics 54: 776–781. 12618910 10.1007/s00251-002-0527-8

[40] O’Reilly V, Zeng SG, Bricard G, Atzberger A, Hogan AE, et al (2011) Distinct and overlapping effector functions of expanded human CD4+, CD8α+ and CD4-CD8α- invariant natural killer T cells. PLoS One 12: e28648. 10.1371/journal.pone.0028648 PMC3236218 22174854

[41] Gansuvd B, Hubbard WJ, Hutchings A, Thomas FT, Goodwin J, et al (2003) Phenotypic and functional characterization of long-term cultured rhesus macaque spleen-derived NKT cells. J Immunol 171: 2904–2911. 12960313 10.4049/jimmunol.171.6.2904

[42] Rout N, Else JG, Yue S, Connole M, Exley MA, et al (2010) Heterogeneity in phenotype and function of CD8+ and CD4/CD8 double-negative Natural Killer T cell subsets in sooty mangabeys. J Med Primatol 39: 224–234. 20618588 10.1111/j.1600-0684.2010.00431.x PMC2904642

